# 33 Real World Cell Viability in Cell Spray Suspension

**DOI:** 10.1093/jbcr/iraf019.033

**Published:** 2025-04-01

**Authors:** Sigrid Blome-Eberwein, Caitlin Stoudt, Hamed Amani, Sakura Helm, Kyle Shaak

**Affiliations:** Lehigh Valley Health Network; Lehigh Valley Health Network; Lehigh Valley Health Network; Lehigh Valley Health Network; Lehigh Valley Health Network

## Abstract

**Introduction:**

Autologous epithelial cell spray, prepared with a commercial kit, is now widely used in American Burn Centers in extensive second and third degree burns where donor skin may be scarce. However, the cell viability and yield of the autologous skin suspension has not been assessed in a real-world setting and there is limited data on patient age and other demographics that may influence the number of viable cells in the suspension. The purpose of this IRB approved study was to evaluate the cell viability of an autologous skin cell suspension in a variety of age groups and Fitzpatrick skin type individuals.

**Methods:**

Patients in our center with burn wounds scheduled to receive split thickness skin graft were consented to participate in the study. Discarded pieces of skin graft were processed immediately, using skin-cell suspension preparation kits according to manufacturer instructions, and processed cells were suspended in 1 mL of buffer solution per cm2 of skin. Cell yield and viability was then measured, partially by manual count in a hemocytometer and partially in an automated cell counter. Three aliquots of each suspension were counted. All data was collected in an online database and analyzed using SPSS statistical software.

**Results:**

64 skin samples from 55 different patients were processed. Patients ranged from 1-86 years old, 40 male, 24 female, 0.25-70.5% total body surface area (TBSA, 7.8% mean), 68.8% Caucasian, 9.4% Black, 21.8% multiracial with Fitzpatrick skin types I-VI. The cell suspensions contained an average of 1,899,367 cells/mL with an average of 785,254 live cells/mL (standard deviation 617,118 cells/mL). There was a wide range of both, cell count and viability across all samples. The average viability across all samples was 41.34% (standard deviation 14.5%). The viability of cell suspensions varied greatly independently of patient age, % TBSA or comorbidities.

**Conclusions:**

The yields of cell suspensions varied greatly and no correlation between patient age or TBSA or different users preparing the cell-suspensions with skin from the same patient was found. Our data is consistent with FDA submitted data from abdominoplasty specimens.

**Applicability of Research to Practice:**

Immediate

**Funding for the Study:**

Institutional foundation funding

